# Post-weaning infant-to-mother bonding in nutritionally independent female mice

**DOI:** 10.1371/journal.pone.0227034

**Published:** 2020-01-15

**Authors:** Stijn Stroobants, John Creemers, Guy Bosmans, Rudi D’Hooge

**Affiliations:** 1 Laboratory of Biological Psychology, KU Leuven, Leuven, Belgium; 2 mINT Behavioral Phenotyping Facility, KU Leuven, Leuven, Belgium; 3 Laboratory of Biochemical Neuroendocrinology, KU Leuven, Leuven, Belgium; 4 Parenting and Special Education Research Unit, KU Leuven, Leuven, Belgium; Radboud University Medical Centre, NETHERLANDS

## Abstract

Infant-parent attachment is highly selective and continues beyond essential care in primates, most prominently in humans, and the quality of this attachment crucially determines cognitive and emotional development of the infant. Altricial rodent species such as mice (*Mus musculus*) display mutual recognition and communal nursing in wild and laboratory environments, but parental bonding beyond the nursing period has not been reported. We presently demonstrated that socially and nutritionally independent mice still prefer to interact selectively with their mother dam. Furthermore, we observed gender differences in the mother-infant relationship, and showed disruption of this relationship in haploinsufficient Nbea^+/-^ mice, a putative autism model with neuroendocrine dysregulation. To our knowledge, this is the first observation of murine infant-to-mother bonding beyond the nursing period.

## Introduction

According to Bowlby’s attachment theory, caregiver attachment is crucial for cognitive and social-affective development in human infants [1]. Children attach securely to responsive caregivers, whereas suboptimal or inconsistent care can lead to insecure attachment that impacts negatively on long-term well-being[2]. Animal models are crucial here, because environmental, genetic or hormonal factors, or experimental therapeutics that influence attachment, often are impossible to study directly in humans [3–4]. Mice are the most suitable or manageable laboratory rodents that could be used in attachment research, since their altricious young depend on extensive nursing and parental protection. Maternal care declines as pups grow older, but is still significant when they approach weaning age [5]. Moreover, dams that are still housed with their pups during the fourth postpartum week gradually switch from nursing-related to non-nursing interactions [6]. This relationship, however, appears to be asymmetric as specific attachment or bonding beyond weaning has been proposed not to occur in the pups [7].

Although mouse studies contributed to our understanding of mother-infant interaction and social behavior in general [4, 8], they mostly focused on maternal deprivation as a correlate of human child neglect or abuse [9–10]. Such deprivation studies emphasized the developmental and social-affective importance of maternal care [11], but failed to identify caregiver recognition and bonding in the pups. Caregiver bonding, as well as infant love for that matter, is deemed to be of broad developmental significance, beyond the gratification of immediate nutritional and protection needs [12]. Mouse pups certainly seem to require parental care. Prolonged or intermittent separation from the dam, especially during the first 2–3 postnatal weeks, causes functional defects in the offspring that persist into adulthood, such as impaired learning and memory [13–15], and altered emotionality, reminiscent of human depression and anxiety [16–17]. These behavioral changes have been attributed to a variety of neuroendocrinological and neurobiological alterations, including dysregulation of the hypothalamic-pituitary-adrenal (HPA) axis [18], hippocampal neuron loss [19], and neuroinflammation [20].

Evidence for filial bonding in post-weaning mouse infants remains inconclusive. Mother-infant bonding is thought to establish in the first postnatal weeks, during which intense reciprocal interaction occurs [21–22]. Pups produce olfactory cues [23], and emit separation-induced ultrasonic vocalizations (USVs) that elicit maternal search and retrieval [24], and that dams discriminate from those of unfamiliar pups [25]. USV rates peak around the end of the first week, and progressively decrease during the second postnatal week [26]. USV emission is potentiated after brief reunion with the dam [27], whereas similar to human infants, calming reactions (including USV reduction) occur during maternal carrying [28]. Developmental decline in USV production coincides with the emergence of crawling and eventually walking [29]. Any need for parental affiliation seems to disappear as pups approach weaning (around P21), start moving around, and acquire nutritional and social independence [30]. Pups at P18 still prefer the odor of their mother over that of a nulliparous female, but fail to discriminate between their mother and other lactating females [31]. More recent studies seemed to confirm that infant mice preferentially interact with their mother in contrast to a nulliparous female [32], and indicate that mice aged P17-21 actually discriminate between their mother and an unfamiliar mother [33]. However, the free-interaction methodology used in these reports cannot exclude that the adult females in fact influenced the interaction.

In the present study, we examined the occurrence of affiliative behavior towards the mother dam in 4-week-old, post-weaning infant mice. We wondered whether or not maternal preference would persist in infants, despite their increasing nutritional independence. We used an adapted social preference procedure that allows assessment of the time mice spent in proximity of their mother in comparison to other social interaction partners [34–35]. It has been shown in rats that mothers interact differently with male and female offspring [36], which could contribute to gender differences in social behavior later in life [37]. Therefore, we also compared social preference profiles between male and female infants. Finally, we assessed the consequences of congenital neuroendocrinological dysregulation on mother-infant bonding in post-weaning infant mice with Neurobeachin (Nbea) haploinsufficiency. Nbea encodes a scaffolding protein involved in exocytosis and synaptic transmission [38], and has been proposed a candidate gene for autism spectrum disorder (ASD) in humans [39–40]. Adult Nbea^+/-^ mice display changes in social behavior[41], and Drosophila rugose mutants (the fly homolog of Nbea) show defects in social interaction [42].

## Methods

### Subjects

Control and Nbea^+/-^ offspring was obtained by crossbreeding haploinsufficient male Nbea^+/-^ mice [41] and female C57BL/6J mice (Elevage Janvier, Le-Genest-Saint-Isle, France) at approximately 4 months of age. Males were housed with one female in standard cages under conventional laboratory conditions (12h light/dark cycle, constant room temperature and humidity, ad libitum access to food and water). Paper shreds were provided as nesting material and enrichment. Males were removed after pregnancy was noted, and future mothers were single housed until the pups were born. All infant mice used in this study were born from primiparous dams, and had been separated from their dam around the age of 4 weeks. Post-weaning offspring was kept in same-sex sibling groups. Behavioral experiments started at postnatal day P28 (effective age at first testing day: 27.91 ± 0.14 days), and performed during the light phase of the activity cycle. Following behavioral testing, juvenile mice were genotyped using PCR on DNA obtained from tail samples. Four experimental groups were compared depending on genotype and gender: control (Nbea^+/+^) males (n = 10), control females (n = 10), Nbea^+/-^ males (n = 11) and Nbea^+/-^ females (n = 12). All procedures were reviewed and approved by the animal ethics committee of our university according to EU directives.

### Assessment of maternal preference in juvenile mice

**Fig 1:**
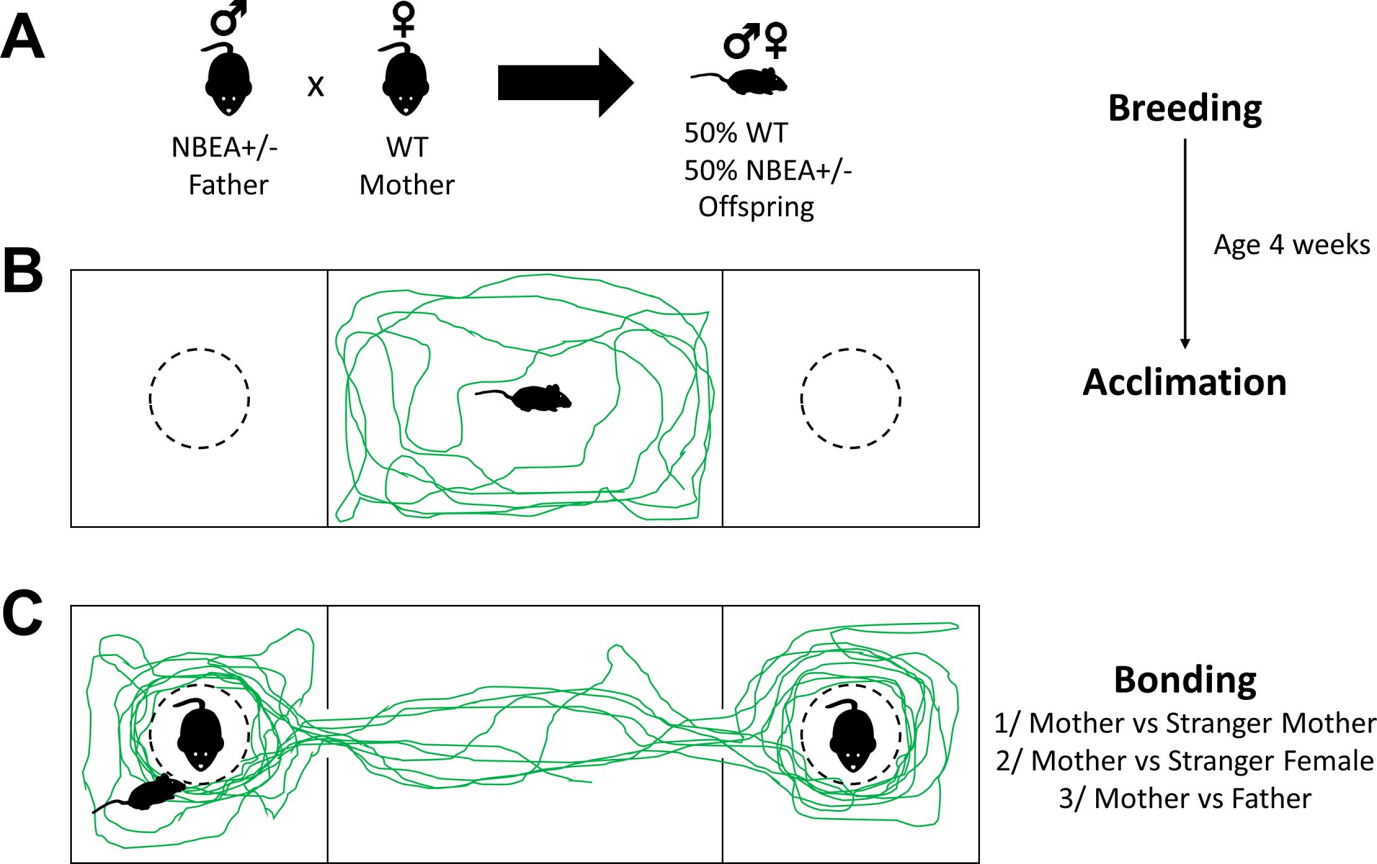
Overview of experimental design. (A) Control and Nbea^+/-^ offspring was obtained by crossbreeding haploinsufficient male Nbea^+/-^ mice and female C57BL/6J mice. All offspring was nursed by control mothers. (B-C) Pups were separated from their dam when they reached the age of 4 weeks. Behavioral experiments started the subsequent day. We implemented a three-chamber approach reminiscent of adult social approach experiments. Following 5 min acclimation in the central chamber, maternal preference was evaluated by comparing the preference of pups to spend time with their mother in comparison to an unfamiliar mother (day 1). During subsequent days, preference for maternal proximity was also assessed in comparison to another adult female (day 2) and the father (day 3).

Maternal preference was evaluated by comparing the preference of infant mice to spend time with their mother in contrast to other interaction partners. For this purpose, we implemented a three-chamber procedure that we and others have used in social approach experiments [34–35]. The test environment consisted of a three-chamber acrylic glass set-up in which chambers were connected by closable doors. The two side chambers (26 x 26 cm) contained cylindrical wired cages (diameter 10 cm, height 11 cm) in which interaction partners could be placed. Preference for maternal proximity was evaluated on 3 consecutive days in post-weaning infant mice at the age of 4 weeks. All animals underwent a minimum of 30 minutes acclimation to the testing room preceding daily experiments. Prior to each experimental trial, an acclimation trial was performed, during which infant mice could explore the central chamber (42 x 26 cm) for 5 min. Following acclimation, the mother and a comparison mouse were placed inside the wired cages. The position of the mother was randomized across subjects. Subsequently, pups were granted access to the side chambers, and their movement was video-tracked during 10 min using ANY-maze^TM^ Video Tracking System software (Stoelting Co., IL, USA). During the experiment, interaction with each respective caged mouse was recorded using the ‘Keys’ press-and-hold function in ANY-maze. In case of tracking inaccuracies, interaction time was manually recalculated using the recorded videos. For analysis of the distance moved parameters, the respective tracks were excluded (9 tracks out of 258). During 3 consecutive days, preferential approach to the mother was assessed in comparison to a stranger mother (day 1), another adult female (day 2) and the father (day 3), which were all unfamiliar to the pups. Fig 1 provides an overview of the experimental design.

### Statistics

Data are presented as means with standard error of the mean (SEM). Group differences were assessed by analysis of variance (ANOVA) procedures, using repeated measure corrections for within-subjects measurements. Between-subjects variables of interest included genotype (control vs Nbea^+/-^) and gender (male vs female). Within-subjects variables included day (1 –stranger mother vs 2 –other female vs 3—father), time (0-2min vs 2-4min vs 4-6min vs 6-8min vs 8-10min) and mother (mother vs other). Sidak adjustments were used for multiple comparisons. Dependent variables included total distance moved, total interaction time, first choice for chamber entry, latency to the first chamber entry, latency to the first interaction after entry as well as the number and duration of interaction phases (an interaction phase refers to the amount of interaction time with a certain stimulus mouse before a switch is made to interact with the other stimulus mouse). The threshold for statistical significance was set at α = 0.05.

## Results

**Fig 2:**
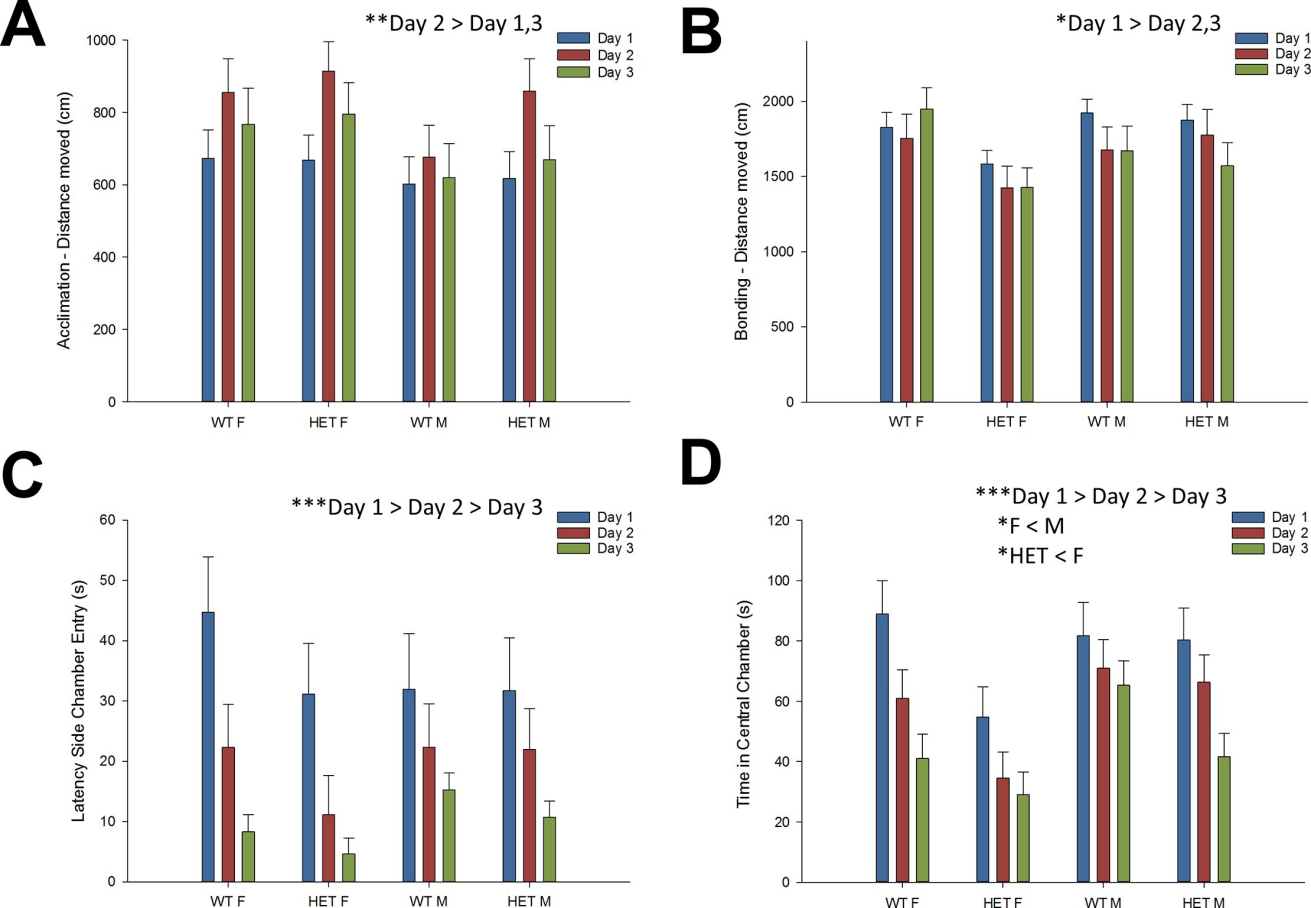
Gender and Nbea genotype influence social exploration, but not general locomotor activity. Distance travelled during the acclimation stages and bonding stages, latency to leave the central chamber and time spent in the central chamber is displayed for day 1 (blue bars), day 2 (red bars) and day 3 (green bars). (A-B) Mice generally travelled more distance on day 2 (vs day 1 & 3) during the acclimation stage and on day 1 (vs day 2 & 3) during the bonding stage. However, there were no significant effects involving gender or genotype on distance travelled during either experimental stage. (C) Mice (in general) became faster to enter the side chambers across subsequent testing days. (D) Females spent less time than males in the central chamber during the bonding stages, as did Nbea^+/-^ mice in comparison to control mice. All data are presented as mean + SEM (control males (n = 10), control females (n = 10), Nbea^+/-^ males (n = 11) and Nbea^+/-^ females (n = 12)). Abbreviations: WT = wildtype control; HET = heterozygote Nbea^+/-^; M = male; F = female. Asterisks indicate significant effects: *p < .05, **p < .01, ***p < .001.

To validate this novel maternal preference test, we first evaluated the distance travelled during the acclimation stages to exclude possible underlying differences in exploratory locomotion. A significant main effect of day indicated that the amount of distance moved during the acclimation stages changed significantly over days (Fig 2A; F = 12.6, *p* < .01). Mice generally travelled most distance during day 2 (prior to mother vs other female) in comparison to day 1 (prior to mother vs stranger mother; *p* < .001) and day 3 (prior to mother vs father; *p* < .01). There were no significant effects involving genotype and/or gender, suggesting comparable ‘non-social’ exploratory activity of the experimental groups. In addition, total distance travelled during the bonding stages was different across days as well (Fig 2B; F = 3.4, *p* < .05). Mice travelled on average more distance during the first day in comparison to day 2 and day 3 (both *p* < .05). Again, no significant effects involving genotype and/or gender were observed on total locomotor activity. Despite decreasing locomotor activity across days, mice became faster to enter one of the side chambers (Fig 2C; F = 15.5, *p* < .001; day 1 > day 2 > day 3). Time spent in the central chamber during bonding stages decreased over days in all groups (Fig 2D; F = 19.6, *p* < .001). However, females generally spent less time in the central chamber (and thus more in the chambers containing the stimulus mice) in comparison to males (F = 5.7, *p* < .05), as did Nbea^+/-^ mice in comparison to control animals (F = 5.1, *p* < .05).

We subsequently performed an overall analysis of the effects of day (1-2-3), time (five 2 min-intervals), stimulus mouse identity (mother & comparison mouse), genotype (control vs Nbea^+/-^) and gender (male-female) on social interaction time during the bonding stages. Combining 3 days of preference testing, we identified significant main effects of gender (F = 5.8, *p* < .05) and genotype (F = 7.5, *p* < .01). Female mice spent more time than males interacting with the stimulus mice, whereas Nbea^+/-^ mice appeared more socially active than controls. A main effect of time (F = 4.8, *p* < .01) was found, reflecting the reduced amount of social interaction during the first 2 min, as mice start from the central chamber, and need to enter one of the side chambers before being able to interact with the stimulus mice. Finally, the main effect of “stimulus mouse identity” indicated that more time was spent investigating the other mice than the mother (across 3 days of testing).

**Fig 3:**
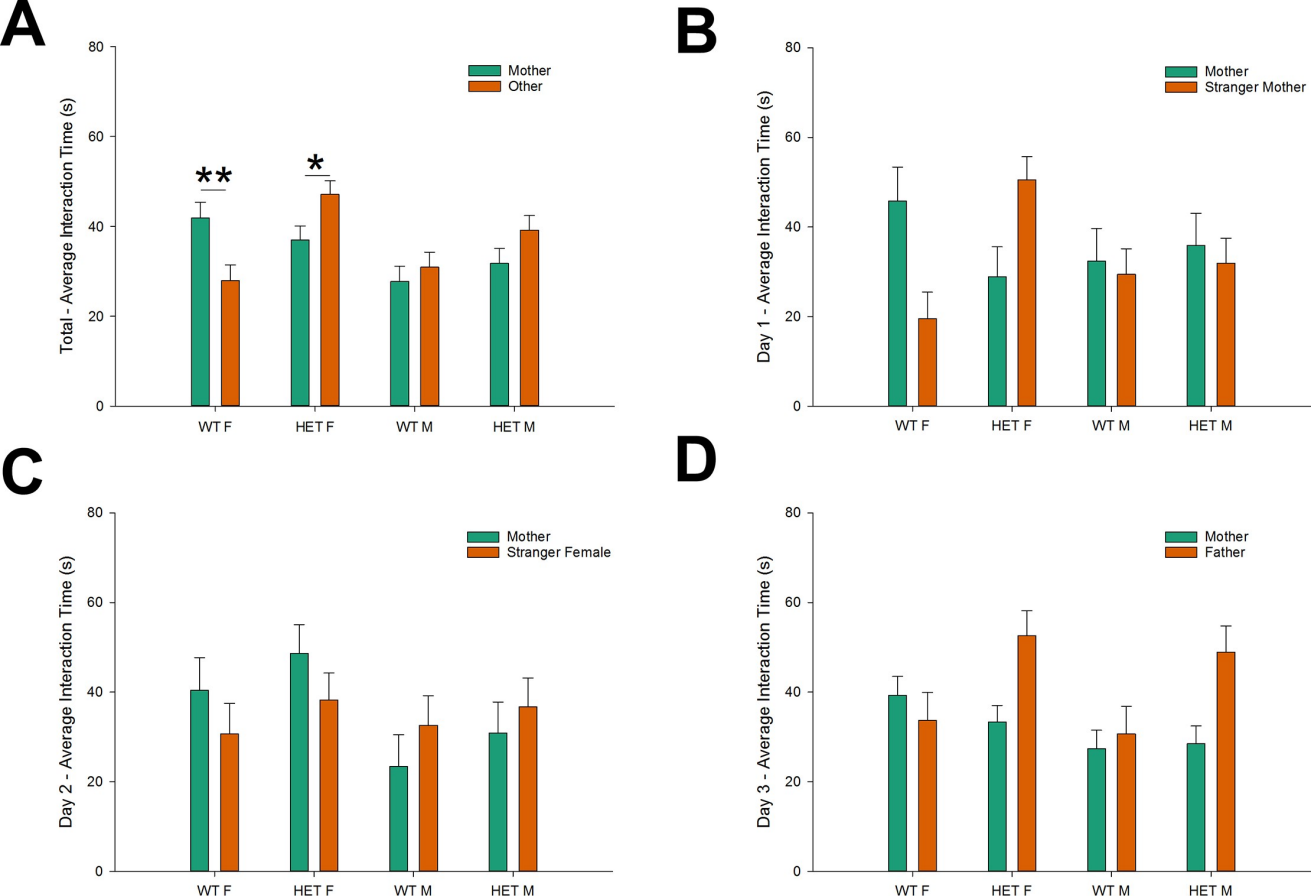
Female control mice seek maternal proximity while Nbea^+/-^ females avoid it. Average interaction time per 2 min with the mother (green bars) and the other stimulus mouse (orange bars) for all days combined and per testing day. (A) Female controls significantly preferred contact with their mother in comparison to other stimulus mice, while female Nbea^+/-^ mice showed a significant preference for other mice in comparison to their mother. A similar trend was observed Nbea^+/-^ males, while male control mice showed similar interaction levels with their mother and the stimulus mice. (B) The aforementioned trends were most clearly reflected during the simultaneous presentation of the mother and the stranger mother on day 1. Plots of the comparison with another female (C) and the father (D) show changes of preference patterns across testing days. All data are presented as mean + SEM (control males (n = 10), control females (n = 10), Nbea^+/-^ males (n = 11) and Nbea^+/-^ females (n = 12)). Abbreviations: WT = wildtype control; HET = heterozygote Nbea^+/-^; M = male; F = female. Asterisks indicate significance of difference: *p < .05, **p < .01.

However, the amount of social interaction, and preference for maternal v. non-maternal contact proved to depend to a certain extent on all other included factors, as multiple significant interaction effects were observed: stimulus mouse identity x genotype (F = 10.1, *p* < .01), stimulus mouse identity x genotype x gender (F = 5.1, *p* < .05) and day x time x stimulus mouse identity x genotype (F = 2.5, *p* < .05). The stimulus mouse identity x genotype x gender interaction indicates that the overall preference for maternal contact differs between Nbea^+/-^ and control mice, depending on their gender. We therefore compared specific interaction times between different combinations of gender and genotype (Fig 3A). Female controls significantly preferred contact with their mother in contrast to other stimulus mice (*p* < .01), and spent significantly less time investigating the other stimulus mice in comparison to female Nbea^+/-^ mice (*p* < .001). Furthermore, female Nbea^+/-^ mice showed a significant preference for other mice in comparison to their mother (*p* < .05), with a similar trend being observed in male Nbea^+/-^ mice (*p* = .10). Male control mice on the other hand showed on average similar social interaction levels with the mother and other stimulus mice (*p* = .49), and interacted significantly less with the mother in comparison to female controls (*p* < .01). These specific general trends were most clearly reflected during the simultaneous presentation of the mother and the stranger mother (Fig 3B), with plots of subsequent presentations (Fig 3C and 3D) hinting at changes of preference patterns across testing days.

**Fig 4:**
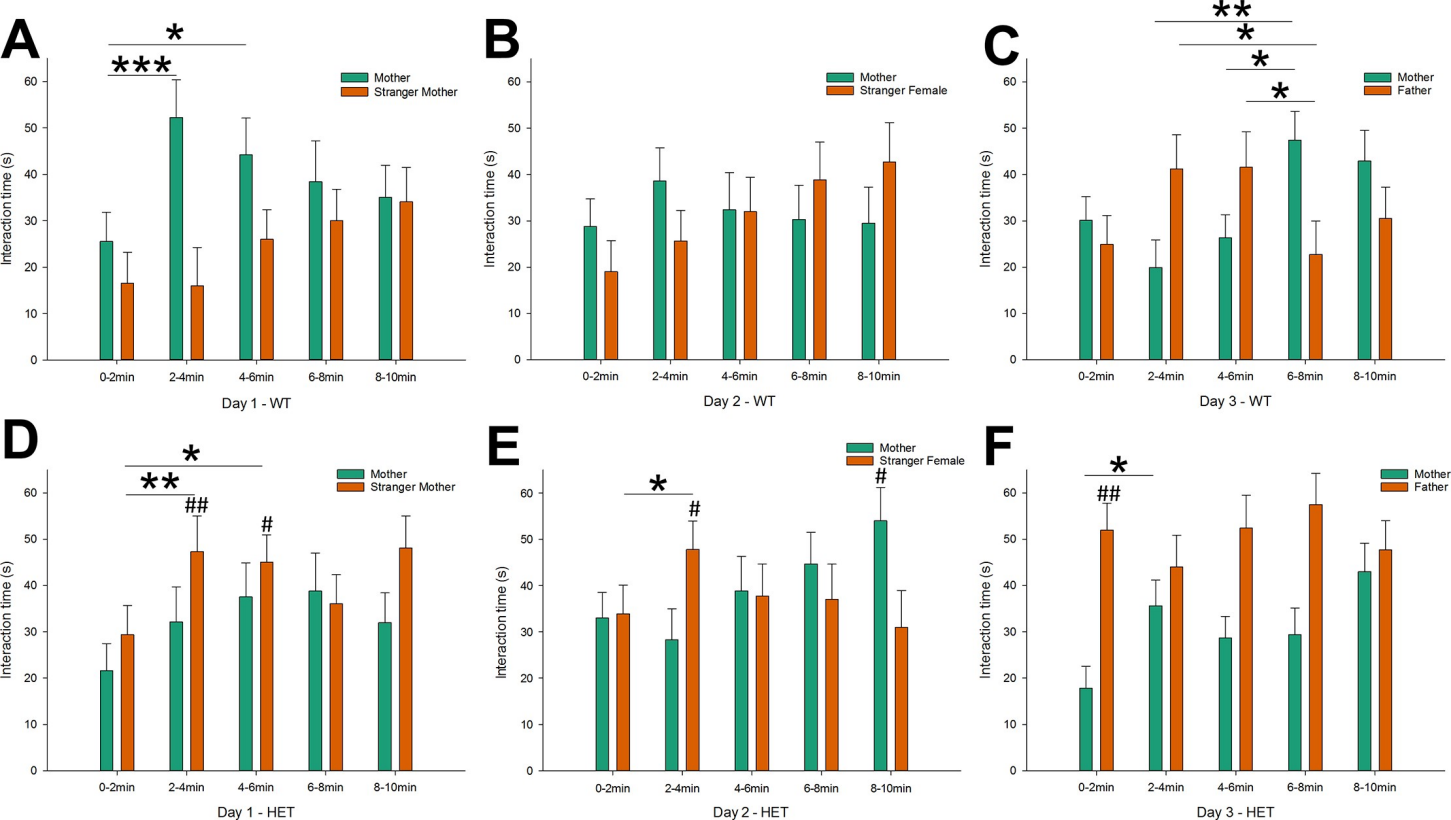
Nbea^+/-^ mice show reduced maternal proximity seeking. Interaction time per 2 min with the mother (green bars) and other stimulus mice (orange bars) per testing day in control mice and Nbea^+/-^ (both genders combined). (A) Control mice show an initial preference to spend time with the mother in comparison to a stranger mother. A similar pattern was observed for the comparison with the stranger female (B), while preference was delayed during the comparison with the father (C). In contrast, Nbea^+/-^ mice showed an initial preference to spend time with the stranger mother in comparison to their own mother (D). Similar trends were observed comparing the mother with another female (E), while they strongly preferred the father on the last testing day (F). All data are presented as mean + SEM (control mice (n = 20), Nbea^+/-^ mice (n = 23)). Abbreviations: WT = wildtype control; HET = heterozygote Nbea^+/-^. Asterisks indicate significance of change between time intervals (only significant differences between intervals with 0 or 1 interval(s) in between them are shown): *p < .05, **p < .01, ***p < .001. Number signs indicate significance of difference with WT values: ^#^*p* < .05, ^##^*p* < .01.

Indeed, the aforementioned day x time x stimulus mouse identity x genotype interaction indicated different maternal preference patterns in Nbea^+/-^ and control mice, depending on stimulus mouse identity. When mice had the choice between the mother and a stranger mother (day 1), control mice showed a strong increase in interaction with the mother from the first to the second interval (Fig 4A; *p* < .001), which persisted during the third interval (*p* < .05). In contrast, Nbea^+/-^ mice showed an increased interaction with the stranger mother from the first to the second (Fig 4D; *p* < .01) and third interval (*p* < .05), during which they spent significantly more time with the stranger mother in comparison to control mice (*p* < .01 & *p* < .05, respectively). Meanwhile, their interaction with the mother also increased over time, but more slowly and less pronounced (interval 1 v. 4, *p* < .05).

During the second day, when mice had the choice between their mother and another female, the interaction profile of control mice was reminiscent of their behavior during the first day (Fig 4B). They showed a similar but less obvious increase of interaction with the mother between the first and second interval, and a similar but more pronounced increase of interaction with the comparison mouse (the other female) across time (interval 1 v. 5, *p* < .05). Nbea^+/-^ mice again showed a reversed profile (Fig 4E), with an increased interaction with the other female from the first to the second interval (*p* < .05), during which they spent more time with the other female than control mice (*p* < .05). Again, they showed increasing interaction with the mother across time (interval 5 v. interval 1&2, *p* < .05).

When mice subsequently had the choice between their mother and their father, they showed similar interaction times with both mice, before interaction with the father increased during the second and third interval (*p* < .05 vs interval 4). Interaction with the mother increased again during the final intervals. Control mice spent more time with the mother in the fourth interval in comparison to all previous intervals (Fig 4C; *p* < .05 or *p* < .01), which persisted in the fifth interval (interval 5 vs interval 2, *p* < .05). In contrast, Nbea^+/-^ mice showed an immediate, strong preference to spent time with the father in the first interval (Fig 4F; father v. mother, *p* < .01), during which they interacted more with the father than control mice (*p* < .01). This preference for the father persisted throughout the experiment (interval 3 & interval 4, father v. mother p < .05), despite increased interest in the mother across time (interval 2 & 5 vs interval 1: *p* < .05 & *p* < .01, respectively).

Across the 3 days of testing, it therefore appeared that the early preference of control mice for spending time with the mother decreased (interval 2: day 1 vs day 3, *p* < .01), in favor of spending time with the comparison mouse (interval 2: day 1 vs day 3, *p* < .05). Nbea^+/-^ mice already showed increased interaction with the comparison mouse (the stranger mother) on day 1, which was less pronounced during the second day, but further intensified when the father was present (interval 1 & 4: day 3 vs day 1, *p* < .01). Notably, male and female data were combined to describe the day x time x stimulus mouse identity x genotype interaction (Fig 4). Time-dependent data for all conditions is depicted in S1 Fig (females) and S2 Fig (males) to illustrate the influence of gender. After describing the major effects in the data across days, we further detailed the patterns of maternal proximity seeking. We focused on the first and putatively most relevant comparison (mother v. stranger mother), as the preference for maternal proximity of control mice was most clearly distinguishable in this configuration and/as there was no previous exposure of the offspring to the apparatus or the mother.

**Fig 5:**
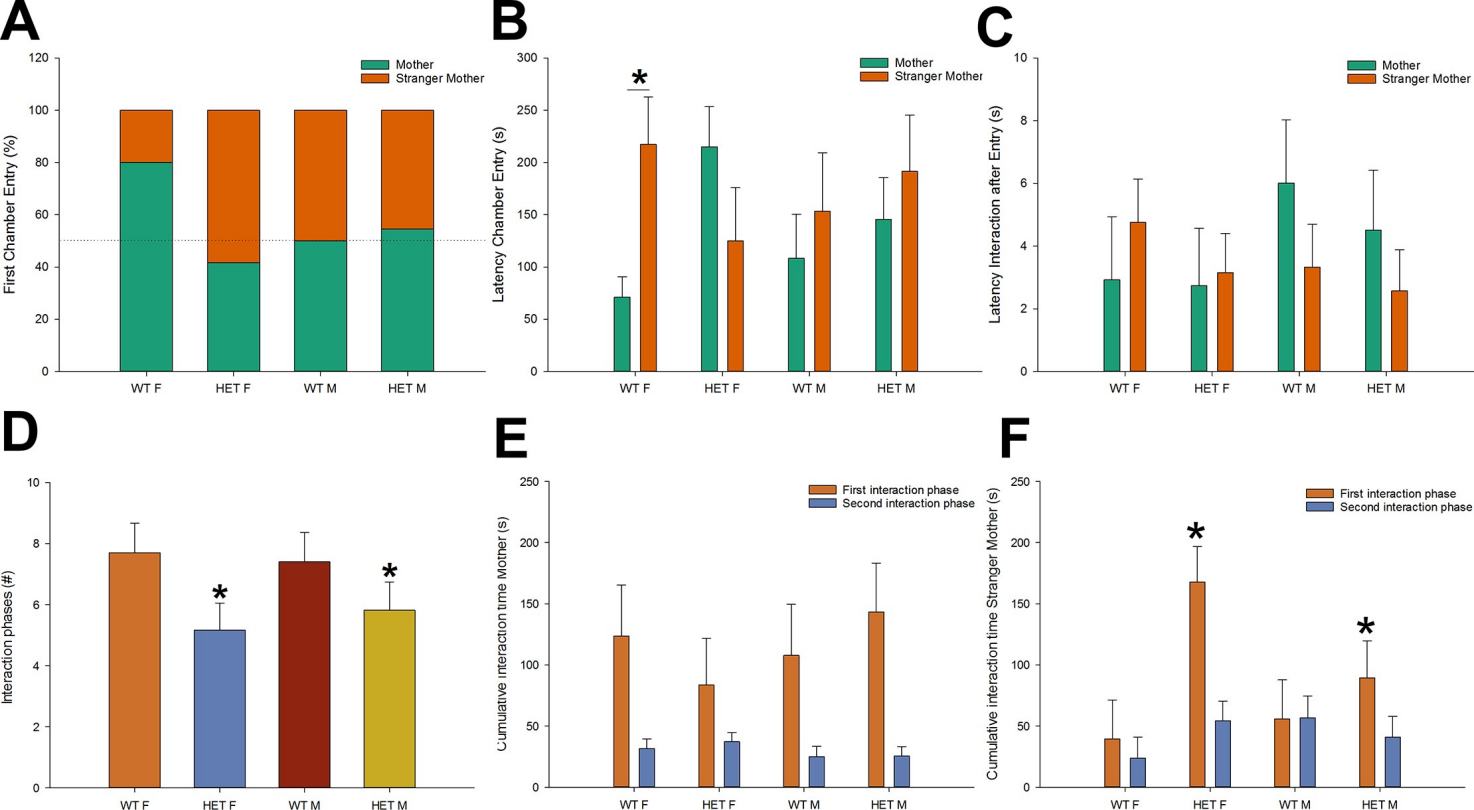
Specification of maternal proximity seeking patterns during comparison with the stranger mother. (A) Female control mice generally chose to enter the chamber of the mother first, in contrast to males and Nbea^+/-^ mice, (B) which correlated to the fact that they showed lower entry latencies for the chamber of the mother in comparison to the stranger mother. (C) There was no influence of genotype or gender on the time to approach the stimulus mothers after the chambers were entered. (D) Male and female NBEA+/- mice showed a reduced amount of interaction phases in comparison to control mice, meaning they switched less between visiting the mother and the stranger mother. (E) The first interaction with the mother was of similar duration in all experimental groups, meaning that mice showed no differences in the amount of interaction upon first contact with the mother before they switched to the stranger mother. In addition, all groups showed a similar decline of cumulative interaction duration from the first to the second interaction phase. (F) Nbea^+/-^mice showed more interaction with the stranger mother during the first interaction phase (before they switched to the mother), meaning control mice switched faster to or back to their own mother. The duration of the second interaction with the stranger mother was similar between the experimental groups. All data are presented as mean + SEM (control males (n = 10), control females (n = 10), Nbea^+/-^ males (n = 11) and Nbea^+/-^ females (n = 12)). Abbreviations: WT = wildtype control; HET = heterozygote Nbea^+/-^; M = male; F = female. Asterisks indicate significance of difference: *p < .05.

Following habituation, the mother and the stranger mother were placed in the side chambers on the first day of testing. Pups remained in the central chamber until the sliding doors were opened. At this point, they had the choice of entering one of the side chambers (Fig 5A). While the majority of control females chose to enter the chamber of the mother (80%), control males and female/male Nbea^+/-^ mice initially entered the chamber of the mother close to chance level (42–55%). This observation related to the fact that control females displayed lower entry latencies for the chamber of the mother in comparison to the stranger mother (Fig 5B; t = 2.2, *p* < .05), whereas other groups showed no significant differences between these latencies. We subsequently evaluated the initial approach to the mother or the stranger mother. For this purpose, we subtracted the chamber entry latency from the latency to first interaction. This measure showed no significant differences between the mother and the stranger mother, nor between the different genotype and gender groups (Fig 5C). This suggests that mice showed no specific reluctance or eagerness to interact directly with the different mothers after they entered the side chambers.

**Fig 6:**
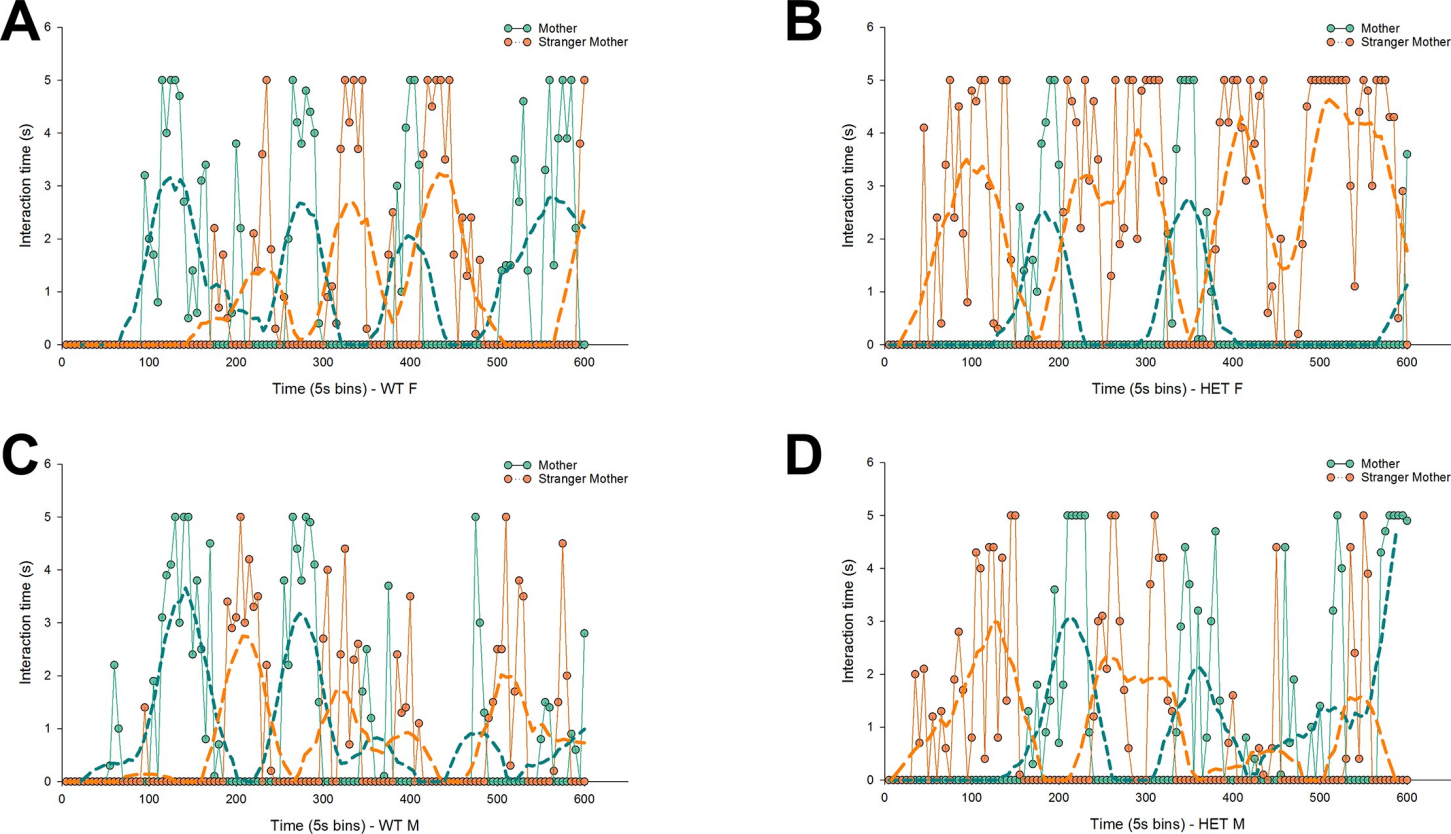
Representative interaction plots illustrate gender and Nbea genotype influences on maternal proximity seeking. Interaction time per 5s with the mother (green lines) and the stranger mother (orange lines) per testing day in a female control mouse (A), a female Nbea^+/-^ mouse (B), a male control mouse (C) and a male Nbea^+/-^ mouse (D). Data were subjected to negative exponential smoothing for clearer visualization of typical temporal interaction patterns observed in the different experimental groups (broken green and orange lines). Control mice show (initial) maternal preference, while Nbea mice show maternal avoidance. Both patterns are most clearly observed in female mice.

Following the first choice, mice typically alternated visits to both side chambers. To further detail possible patterns in maternal proximity seeking, we determined the interaction phases for each mouse, defined by the amount of interaction time with a certain stimulus mouse before a switch is made to interact with the other stimulus mouse. Male as well as female Nbea^+/-^ mice showed a reduced amount of interaction phases in comparison to control mice (Fig 5D: F = 4.9, *p* < .05). There were no significant effects of gender. Subsequently we compared the duration of the first interaction involving the mother and the stranger mother, independent of which stimulus mouse was visited first. There appeared to be no significant effects involving the duration of the first interaction phase with the mother (Fig 5E). However, there was a significant effect of genotype on the duration of the first phase of interaction with the stranger mother (Fig 5F; F = 6.9, *p* < .05). Nbea^+/-^ mice showed increased interaction with the stranger mother, meaning control mice switched faster to or back to their own mother. Following the initial interactions of the offspring with both stimulus mice, the second (and later) interaction phases proved significantly shorter (Fig 5E–5F; F = 22.1, *p* < .001) and the duration showed no persistent influences of genotype (or gender). Fig 6 includes representative interaction plots for all experimental groups, which represent the amount of interaction time with the mother and the stranger mother in 5s intervals. For every group, a mouse was selected that showed minimal deflection of the average values. Subsequently, these data were subjected to negative exponential smoothing, which allowed for a clearer visualization of typical temporal interaction patterns observed in the different experimental groups (Fig 6A–6D). Female control mice showed a higher probability to interact first with the mother, with control mice in general interacting relatively more during their first confrontation with the mother, whereas the reverse was true in Nbea^+/-^ mice (most notably in females).

## Discussion

Mice display communal nursing in wild and laboratory settings, and it has long been supposed that specific bonding of pups with their mother dam may not exist in this species [7]. Assessment of maternal care during the first 2 postnatal weeks appeared to be consistent with this notion of asymmetric mother-infant bonding in mice [21]. Recent studies that used free social interaction procedures could not exclude the influence of the adult interaction partner [32–33]. Our present results indicate that specific maternal preference does occur in post-weaning females, but not in males. Female infants approached their mother faster than a stranger mother, and also spent more time interacting with their mother in comparison to another mother or other mice in general. Thus, this is the first observation of a filial bond, persisting in pups beyond the age of nutritional dependence. Notably, simultaneously presenting a familiar and an unfamiliar mouse to an infant was expected to increase exploration of the unfamiliar mouse [43]. However, our observations rather indicate that the mother is recognized by the infant, and that the need for parental proximity overrides the natural tendency to explore novel (social) stimuli. Furthermore, touching base with the mother seemed to facilitate subsequent social exploration.

Offspring gender is one of many factors influencing maternal care, which has important consequences for neurobehavioral development [11]. Rat mothers spend more time nursing and licking/grooming male than female pups [36, 44]. Furthermore, maternal deprivation affects brain development and function differently in male and female rodents [45–48]. Post-weaning decline in maternal care coincides with an increase in social interaction between siblings[49]. Interestingly, female infants were shown to be more social towards siblings than males, whereas males were more social towards non-sibling interaction partners than females [50]. This difference in affiliative behaviors between females and males might relate to the presently observed increased social interaction with their mother than with an unfamiliar mother. These observations also demonstrate a persisting social bond with mother and female littermates. This apparent sexually dimorphic evolution of mother-infant bonding in mice marks the transition into adulthood, during which female mice generally maintain affiliative relationships with same-gender conspecifics. In contrast, adult males develop territorial behaviors in the context of dominance hierarchies[49].

Research on neural circuits underlying mother-infant bonding has mainly focused on affiliative maternal behavior [51]. Neuropeptides oxytocin and vasopressin in interaction with the dopaminergic reward system have been shown to play a role in the expression of maternal behaviors [52]. Filial attachment and other affiliative social behaviors are considered to share many psychobiological mechanisms [53]. The neural pathways involved in attachment in infants are unclear, but vasopressin and oxytocin could also play a role here since they are also present in the developing brain. Neonatal social behavior is influenced by the activity levels of both neuropeptides [54–55]. Activation of the infant oxytocin system is provoked by the sensory experiences of maternal engagement [56], which could control reciprocal bonding behaviors between mother and infant [22].

We observed reduced maternal proximity seeking in infant Nbea^+/-^ mice. Similar path lengths across conditions indicated that these differences were not related to changes in locomotor activity. Female Nbea^+/-^ mice spent significantly more time interacting with the other stimulus mice than with their own mother, actually rather avoiding their mother. Male Nbea ^+/-^ mice showed similar interaction levels with their mother and female stimulus mice, comparable to male controls, but tended to approach the male more. Knockdown of Nbea triggers enhanced secretion of dense-core secretory granules in neuroendocrine cells [39]. Hence, dysregulated hormone/neuropeptide release could be related to the changes in filial bonding in post-weaning Nbea^+/-^ mice. Additionally, changes in offspring responses might have influenced maternal behaviors[57], further disturbing the regular, oxytocin-mediated loop of mother-infant contacts.

The neural circuits underlying early infant attachment could thus be disturbed in Nbea^+/-^ mice. Nbea has been proposed as an autism candidate gene following the identification of Nbea gene alterations in ASD patients[38]. Adult female Nbea^+/-^ mice show autism-like behaviors such as increased self-grooming and reduced sociability in the three-chamber test. However, they are able to recognize a previously encountered conspecific and prefer to spend time with an unfamiliar mouse similar to controls[41]. The disrupted mother-infant relationship we presently observed may be part of the neurodevelopmental cascade that eventually leads to the adult phenotype, considering the importance of mother-infant bonding. However, the role of mother-infant bonding in ASD is still poorly understood[58]. Despite atypical reciprocal social interactions, children with ASD might still be able to develop secure attachments to their primary caregiver[59], although reports are conflicting [60]. Reduced cognitive ability in addition to ASD core symptoms could be an important factor [61]. Nbea^+/-^ mice show impaired neurocognitive performance coinciding with increased hippocampal long-term potentiation and phosphorylation of the signaling protein CREB [41]. Notably, phosphorylated CREB expression is strongly linked to primary maternal odor learning [62].

Human attachment tends to become increasingly stable towards adulthood, allowing more insight into the outcome of attachment development at this stage[63]. We specifically chose to evaluate infant-to-mother bonding at the end of the fourth postnatal week, a time point considered beyond extensive maternal care. As a consequence, laboratory weaning was performed later than often practiced. Laboratory weaning of mice is typically brusquely performed around p21, guided by practical reasons for colony management. Earlier weaning, often performed at the end of postnatal week 2, constitutes a maternal deprivation manipulation, leading to detrimental effects on neurobiological and behavioral functions in later life [9]. Not much is known about effects of ‘late weaning’ on offspring development. Late weaning murine offspring were shown to display neural and behavioral changes, which however are typically not assessed before mice approach adulthood [6, 64]. It was also shown in mice that effects of early life experiences may become more visible in full adulthood in comparison to adolescence [65]. Furthermore, late weaning mice were shown to initially develop higher body weights, which disappeared and reversed over time [64].

Weaning is a gradual process and ‘late weaning’–as performed here–could actually better represent the natural cascade of this phase in the transition to adulthood. In future studies, it would however be interesting to systematically assess maternal proximity seeking at different weaning ages, such that the extinction of infant-to-mother bonding behavior could be mapped depending on the age and/or stage of maturation of the infants. In addition, we relied upon observations in earlier studies to estimate characteristics of maternal behavior in relationship to the age of the offspring. Future experiments preferably include assessments of maternal care patterns as well, such that these can be directly linked to maternal proximity seeking in infants.

In conclusion, our results indicate that post-weaning female mice, but not males, still prefer their mother to another postpartum female. This is the first demonstration of gender-specific mother bonding beyond weaning. Furthermore, we showed that this relationship is disrupted in Nbea^+/-^ mice, a putative ASD mouse model with neuroendocrine dysregulation. The reported quantification of filial bonding in infant mice can be used to study the mechanisms of attachment-like behaviors and their importance in cognitive and emotional development.

## Supporting information

S1 Fig Female control mice show persistent maternal preference in contrast to persistent maternal avoidance in Nbea^+/-^females. Interaction time per 2 min with the mother (green bars) and other stimulus mice (orange bars) per testing day in female control mice and female Nbea^+/-^ mice. (A-C) Female controls initially prefer contact with their mother in comparison to other stimulus mice. This marked preference declines across testing days. (D-F) Female Nbea^+/-^ mice showed increased interaction with the stranger mother in comparison with their own mother. Reduced interaction with the mother was less pronounced during the second day, but was intensified when the father was presented on the third day of testing. All data are presented as mean + SEM (control females (n = 10) and Nbea^+/-^ females (n = 12)). Abbreviations: WT = wildtype control; HET = heterozygote Nbea^+/-^.
(TIF)

S2 Fig Male control mice show very limited maternal preference in contrast to increasing maternal avoidance in Nbea^+/-^ males. Interaction time per 2 min with the mother (green bars) and other stimulus mice (orange bars) per testing day in male control mice and male Nbea^+/-^ mice. (A-C) Male controls show an initial increase in contact with their mother in comparison to the stranger mother, but maternal preference quickly declines and is not observed again in subsequent days.(D-F) Male Nbea^+/-^ mice do not show maternal preference in comparison with the stranger mother. Increasing maternal avoidance was observed in subsequent comparisons with the other female and the father. All data are presented as mean + SEM (control males (n = 10), Nbea^+/-^ males (n = 11)). Abbreviations: WT = wildtype control; HET = heterozygote Nbea^+/-^.
(TIF)

S1 File Dataset. (XLSX)

## References

[1] Bowlby J. Attachment and loss: Vol. 1. Attachment. New York, NY: Basic Books; 1969.

[2] McCrory E, De Brito SA, Viding E. The link between child abuse and psychopathology: a review of neurobiological and genetic research. Journal of the Royal Society of Medicine. 2012;105(4):151–6. Epub 2012/04/26. 10.1258/jrsm.2011.110222 22532655 PMC3343716

[3] Esposito G, Setoh P, Shinohara K, Bornstein MH. The development of attachment: Integrating genes, brain, behavior, and environment. Behavioural brain research. 2017;325(Pt B):87–9. Epub 2017/03/23. 10.1016/j.bbr.2017.03.025 28322913 PMC5860659

[4] Lucion AB, Bortolini MC. Mother-pup interactions: rodents and humans. Frontiers in endocrinology. 2014;5:17 Epub 2014/03/13. 10.3389/fendo.2014.00017 24616713 PMC3935307

[5] Kikusui T, Isaka Y, Mori Y. Early weaning deprives mouse pups of maternal care and decreases their maternal behavior in adulthood. Behavioural brain research. 2005;162(2):200–6. Epub 2005/06/23. 10.1016/j.bbr.2005.03.013 . 15970216

[6] Curley JP, Jordan ER, Swaney WT, Izraelit A, Kammel S, Champagne FA. The meaning of weaning: influence of the weaning period on behavioral development in mice. Developmental neuroscience. 2009;31(4):318–31. Epub 2009/06/24. 10.1159/000216543 19546569 PMC2820580

[7] Gubernick DJ. Parent and infant attachment in mammals In Gubernick DJ, Knopfler PH, editors. Parental care in mammals. Boston, MA: Springer; 1981 pp. 243–305.

[8] Stoesz BM, Hare JF, Snow WM. Neurophysiological mechanisms underlying affiliative social behavior: insights from comparative research. Neuroscience and biobehavioral reviews. 2013;37(2):123–32. Epub 2012/11/28. 10.1016/j.neubiorev.2012.11.007 . 23182913

[9] Kikusui T, Mori Y. Behavioural and neurochemical consequences of early weaning in rodents. Journal of neuroendocrinology. 2009;21(4):427–31. Epub 2009/02/12. 10.1111/j.1365-2826.2009.01837.x . 19207810

[10] Tractenberg SG, Levandowski ML, de Azeredo LA, Orso R, Roithmann LG, Hoffmann ES, et al An overview of maternal separation effects on behavioural outcomes in mice: Evidence from a four-stage methodological systematic review. Neuroscience and biobehavioral reviews. 2016;68:489–503. Epub 2016/06/23. 10.1016/j.neubiorev.2016.06.021 . 27328784

[11] Curley JP, Champagne FA. Influence of maternal care on the developing brain: Mechanisms, temporal dynamics and sensitive periods. Frontiers in neuroendocrinology. 2016;40:52–66. Epub 2015/12/01. 10.1016/j.yfrne.2015.11.001 26616341 PMC4783284

[12] Landers MS, Sullivan RM. The development and neurobiology of infant attachment and fear. Developmental neuroscience. 2012;34(2–3):101–14. Epub 2012/05/11. 10.1159/000336732 22571921 PMC3593124

[13] Wang L, Jiao J, Dulawa SC. Infant maternal separation impairs adult cognitive performance in BALB/cJ mice. Psychopharmacology. 2011;216(2):207–18. Epub 2011/02/19. 10.1007/s00213-011-2209-4 . 21331521

[14] Chocyk A, Przyborowska A, Makuch W, Majcher-Maslanka I, Dudys D, Wedzony K. The effects of early-life adversity on fear memories in adolescent rats and their persistence into adulthood. Behavioural brain research. 2014;264:161–72. Epub 2014/02/11. 10.1016/j.bbr.2014.01.040 . 24508235

[15] Nishi M, Horii-Hayashi N, Sasagawa T. Effects of early life adverse experiences on the brain: implications from maternal separation models in rodents. Frontiers in neuroscience. 2014;8:166 Epub 2014/07/06. 10.3389/fnins.2014.00166 24987328 PMC4060417

[16] Lee JH, Kim HJ, Kim JG, Ryu V, Kim BT, Kang DW, et al Depressive behaviors and decreased expression of serotonin reuptake transporter in rats that experienced neonatal maternal separation. Neuroscience research. 2007;58(1):32–9. Epub 2007/02/15. 10.1016/j.neures.2007.01.008 . 17298851

[17] Shin SY, Han SH, Woo RS, Jang SH, Min SS. Adolescent mice show anxiety- and aggressive-like behavior and the reduction of long-term potentiation in mossy fiber-CA3 synapses after neonatal maternal separation. Neuroscience. 2016;316:221–31. Epub 2016/01/07. 10.1016/j.neuroscience.2015.12.041 . 26733385

[18] Aisa B, Tordera R, Lasheras B, Del Rio J, Ramirez MJ. Effects of maternal separation on hypothalamic-pituitary-adrenal responses, cognition and vulnerability to stress in adult female rats. Neuroscience. 2008;154(4):1218–26. Epub 2008/06/17. 10.1016/j.neuroscience.2008.05.011 . 18554808

[19] Fabricius K, Wortwein G, Pakkenberg B. The impact of maternal separation on adult mouse behaviour and on the total neuron number in the mouse hippocampus. Brain structure & function. 2008;212(5):403–16. Epub 2008/01/18. 10.1007/s00429-007-0169-6 18200448 PMC2226080

[20] Gracia-Rubio I, Moscoso-Castro M, Pozo OJ, Marcos J, Nadal R, Valverde O. Maternal separation induces neuroinflammation and long-lasting emotional alterations in mice. Progress in neuro-psychopharmacology & biological psychiatry. 2016;65:104–17. Epub 2015/09/19. 10.1016/j.pnpbp.2015.09.003 . 26382758

[21] Mogi K, Nagasawa M, Kikusui T. Developmental consequences and biological significance of mother-infant bonding. Progress in neuro-psychopharmacology & biological psychiatry. 2011;35(5):1232–41. Epub 2010/09/08. 10.1016/j.pnpbp.2010.08.024 . 20817069

[22] Nagasawa M, Okabe S, Mogi K, Kikusui T. Oxytocin and mutual communication in mother-infant bonding. Frontiers in human neuroscience. 2012;6:31 Epub 2012/03/01. 10.3389/fnhum.2012.00031 22375116 PMC3289392

[23] Wang Z, Storm DR. Maternal behavior is impaired in female mice lacking type 3 adenylyl cyclase. Neuropsychopharmacology: official publication of the American College of Neuropsychopharmacology. 2011;36(4):772–81. Epub 2010/12/15. 10.1038/npp.2010.211 21150908 PMC3055720

[24] Wohr M, Schwarting RK. Affective communication in rodents: ultrasonic vocalizations as a tool for research on emotion and motivation. Cell and tissue research. 2013;354(1):81–97. Epub 2013/04/12. 10.1007/s00441-013-1607-9 . 23576070

[25] Okabe S, Nagasawa M, Kihara T, Kato M, Harada T, Koshida N, et al Pup odor and ultrasonic vocalizations synergistically stimulate maternal attention in mice. Behavioral neuroscience. 2013;127(3):432–8. Epub 2013/04/03. 10.1037/a0032395 . 23544596

[26] Hahn ME, Karkowski L, Weinreb L, Henry A, Schanz N, Hahn EM. Genetic and developmental influences on infant mouse ultrasonic calling. II. Developmental patterns in the calls of mice 2–12 days of age. Behavior genetics. 1998;28(4):315–25. Epub 1998/11/06. 10.1023/a:1021679615792 . 9803024

[27] Shair HN. Parental potentiation of vocalization as a marker for filial bonds in infant animals. Developmental psychobiology. 2014;56(8):1689–97. Epub 2014/06/12. 10.1002/dev.21222 . 24915803

[28] Esposito G, Yoshida S, Ohnishi R, Tsuneoka Y, Rostagno Mdel C, Yokota S, et al Infant calming responses during maternal carrying in humans and mice. Current biology: CB. 2013;23(9):739–45. Epub 2013/04/23. 10.1016/j.cub.2013.03.041 . 23602481

[29] Heyser CJ. Assessment of developmental milestones in rodents. Current protocols in neuroscience. 2004;Chapter 8:Unit 8 18. Epub 2008/04/23. 10.1002/0471142301.ns0818s25 . 18428605

[30] Yoshida S, Esposito G, Ohnishi R, Tsuneoka Y, Okabe S, Kikusui T, et al Transport Response is a filial-specific behavioral response to maternal carrying in C57BL/6 mice. Frontiers in zoology. 2013;10(1):50 Epub 2013/08/16. 10.1186/1742-9994-10-50 23945354 PMC3751433

[31] Breen MF, Leshner AI. Maternal pheromone: A demonstration of its existence in the mouse (Mus musculus). Physiology & behavior. 1977;18(3):527–9. 10.1016/0031-9384(77)90269-4.

[32] Lassi G, Tucci V. Gene-environment interaction influences attachment-like style in mice. Genes, brain, and behavior. 2017;16(6):612–8. Epub 2017/04/20. 10.1111/gbb.12385 . 28421709

[33] Mogi K, Takakuda A, Tsukamoto C, Ooyama R, Okabe S, Koshida N, et al Mutual mother-infant recognition in mice: The role of pup ultrasonic vocalizations. Behavioural brain research. 2017;325(Pt B):138–46. Epub 2016/08/29. 10.1016/j.bbr.2016.08.044 . 27567527

[34] Nadler JJ, Moy SS, Dold G, Trang D, Simmons N, Perez A, et al Automated apparatus for quantitation of social approach behaviors in mice. Genes, brain, and behavior. 2004;3(5):303–14. Epub 2004/09/04. 10.1111/j.1601-183X.2004.00071.x . 15344923

[35] Naert A, Callaerts-Vegh Z, D'Hooge R. Nocturnal hyperactivity, increased social novelty preference and delayed extinction of fear responses in post-weaning socially isolated mice. Brain research bulletin. 2011;85(6):354–62. Epub 2011/04/20. 10.1016/j.brainresbull.2011.03.027 . 21501666

[36] Moore CL, Morelli GA. Mother rats interact differently with male and female offspring. Journal of comparative and physiological psychology. 1979;93(4):677–84. Epub 1979/08/01. 10.1037/h0077599 . 479402

[37] Edelmann MN, Demers CH, Auger AP. Maternal touch moderates sex differences in juvenile social play behavior. PloS one. 2013;8(2):e57396 Epub 2013/03/06. 10.1371/journal.pone.0057396 23460849 PMC3583898

[38] Volders K, Nuytens K, Creemers JW. The autism candidate gene Neurobeachin encodes a scaffolding protein implicated in membrane trafficking and signaling. Current molecular medicine. 2011;11(3):204–17. Epub 2011/03/08. 10.2174/156652411795243432 . 21375492

[39] Castermans D, Volders K, Crepel A, Backx L, De Vos R, Freson K, et al SCAMP5, NBEA and AMISYN: three candidate genes for autism involved in secretion of large dense-core vesicles. Human molecular genetics. 2010;19(7):1368–78. Epub 2010/01/15. 10.1093/hmg/ddq013 . 20071347

[40] Castermans D, Wilquet V, Parthoens E, Huysmans C, Steyaert J, Swinnen L, et al The neurobeachin gene is disrupted by a translocation in a patient with idiopathic autism. Journal of medical genetics. 2003;40(5):352–6. Epub 2003/05/15. 10.1136/jmg.40.5.352 12746398 PMC1735479

[41] Nuytens K, Gantois I, Stijnen P, Iscru E, Laeremans A, Serneels L, et al Haploinsufficiency of the autism candidate gene Neurobeachin induces autism-like behaviors and affects cellular and molecular processes of synaptic plasticity in mice. Neurobiology of disease. 2013;51:144–51. Epub 2012/11/17. 10.1016/j.nbd.2012.11.004 . 23153818

[42] Wise A, Tenezaca L, Fernandez RW, Schatoff E, Flores J, Ueda A, et al Drosophila mutants of the autism candidate gene neurobeachin (rugose) exhibit neuro-developmental disorders, aberrant synaptic properties, altered locomotion, and impaired adult social behavior and activity patterns. Journal of neurogenetics. 2015;29(2–3):135–43. Epub 2015/06/24. 10.3109/01677063.2015.1064916 26100104 PMC4747641

[43] Kaidanovich-Beilin O, Lipina T, Vukobradovic I, Roder J, Woodgett JR. Assessment of social interaction behaviors. Journal of visualized experiments: JoVE. 2011;(48). Epub 2011/03/16. 10.3791/2473 21403628 PMC3197404

[44] Richmond G, Sachs BD. Maternal discrimination of pup sex in rats. Developmental psychobiology. 1984;17(1):87–9. Epub 1984/01/01. 10.1002/dev.420170108 . 6698313

[45] Kikusui T, Kiyokawa Y, Mori Y. Deprivation of mother-pup interaction by early weaning alters myelin formation in male, but not female, ICR mice. Brain research. 2007;1133(1):115–22. Epub 2006/12/23. 10.1016/j.brainres.2006.11.031 . 17184748

[46] Oomen CA, Girardi CE, Cahyadi R, Verbeek EC, Krugers H, Joels M, et al Opposite effects of early maternal deprivation on neurogenesis in male versus female rats. PloS one. 2009;4(1):e3675 Epub 2009/01/31. 10.1371/journal.pone.0003675 19180242 PMC2629844

[47] Bondar NP, Lepeshko AA, Reshetnikov VV. Effects of Early-Life Stress on Social and Anxiety-Like Behaviors in Adult Mice: Sex-Specific Effects. Behavioural neurology. 2018;2018:1538931 Epub 2018/04/06. 10.1155/2018/1538931 29619126 PMC5818933

[48] Xu H, Ye Y, Hao Y, Shi F, Yan Z, Yuan G, et al Sex differences in associations between maternal deprivation and alterations in hippocampal calcium-binding proteins and cognitive functions in rats. Behavioral and Brain Functions. 2018;14(1):10 10.1186/s12993-018-0142-y 29759084 PMC5952636

[49] Arakawa H. Ethological approach to social isolation effects in behavioral studies of laboratory rodents. Behavioural brain research. 2018;341:98–108. Epub 2017/12/31. 10.1016/j.bbr.2017.12.022 . 29287909

[50] Cox KH, Rissman EF. Sex differences in juvenile mouse social behavior are influenced by sex chromosomes and social context. Genes, brain, and behavior. 2011;10(4):465–72. Epub 2011/03/19. 10.1111/j.1601-183X.2011.00688.x 21414140 PMC3107935

[51] Numan M, Young LJ. Neural mechanisms of mother-infant bonding and pair bonding: Similarities, differences, and broader implications. Hormones and behavior. 2016;77:98–112. Epub 2015/06/13. 10.1016/j.yhbeh.2015.05.015 26062432 PMC4671834

[52] Bosch OJ, Neumann ID. Both oxytocin and vasopressin are mediators of maternal care and aggression in rodents: from central release to sites of action. Hormones and behavior. 2012;61(3):293–303. Epub 2011/11/22. 10.1016/j.yhbeh.2011.11.002 . 22100184

[53] Coria-Avila GA, Manzo J, Garcia LI, Carrillo P, Miquel M, Pfaus JG. Neurobiology of social attachments. Neuroscience and biobehavioral reviews. 2014;43:173–82. Epub 2014/04/29. 10.1016/j.neubiorev.2014.04.004 . 24769402

[54] Hammock EA, Law CS, Levitt P. Vasopressin eliminates the expression of familiar odor bias in neonatal female mice through V1aR. Hormones and behavior. 2013;63(2):352–60. Epub 2012/12/25. 10.1016/j.yhbeh.2012.12.006 23261858 PMC4285782

[55] Mogi K, Ooyama R, Nagasawa M, Kikusui T. Effects of neonatal oxytocin manipulation on development of social behaviors in mice. Physiology & behavior. 2014;133:68–75. Epub 2014/05/27. 10.1016/j.physbeh.2014.05.010 . 24857720

[56] Hammock EA. Developmental perspectives on oxytocin and vasopressin. Neuropsychopharmacology: official publication of the American College of Neuropsychopharmacology. 2015;40(1):24–42. Epub 2014/05/28. 10.1038/npp.2014.120 24863032 PMC4262889

[57] Takahashi T, Okabe S, Broin PO, Nishi A, Ye K, Beckert MV, et al Structure and function of neonatal social communication in a genetic mouse model of autism. Molecular psychiatry. 2016;21(9):1208–14. Epub 2015/12/17. 10.1038/mp.2015.190 26666205 PMC4909589

[58] Vivanti G, Nuske HJ. Autism, attachment, and social learning: Three challenges and a way forward. Behavioural brain research. 2017;325(Pt B):251–9. Epub 2016/10/23. 10.1016/j.bbr.2016.10.025 . 27751811

[59] Gernsbacher MA, Dissanayake C, Goldsmith HH, Mundy PC, Rogers SJ, Sigman M. Autism and deficits in attachment behavior. Science. 2005;307(5713):1201–3; author reply -3. Epub 2005/02/26. 10.1126/science.307.5713.1201 15731426 PMC4301420

[60] Rutgers AH, Bakermans-Kranenburg MJ, van Ijzendoorn MH, van Berckelaer-Onnes IA. Autism and attachment: a meta-analytic review. Journal of child psychology and psychiatry, and allied disciplines. 2004;45(6):1123–34. Epub 2004/07/20. 10.1111/j.1469-7610.2004.t01-1-00305.x . 15257669

[61] Sivaratnam CS, Newman LK, Tonge BJ, Rinehart NJ. Attachment and Emotion Processing in Children with Autism Spectrum Disorders: Neurobiological, Neuroendocrine, and Neurocognitive Considerations. Review Journal of Autism and Developmental Disorders. 2015;2(2):222–42. 10.1007/s40489-015-0048-7

[62] Raineki C, De Souza MA, Szawka RE, Lutz ML, De Vasconcellos LF, Sanvitto GL, et al Neonatal handling and the maternal odor preference in rat pups: involvement of monoamines and cyclic AMP response element-binding protein pathway in the olfactory bulb. Neuroscience. 2009;159(1):31–8. Epub 2009/01/14. 10.1016/j.neuroscience.2008.12.012 . 19138731

[63] Fraley RC, Vicary AM, Brumbaugh CC, Roisman GI. Patterns of stability in adult attachment: an empirical test of two models of continuity and change. Journal of personality and social psychology. 2011;101(5):974–92. Epub 2011/06/29. 10.1037/a0024150 . 21707199

[64] Richter SH, Kastner N, Loddenkemper DH, Kaiser S, Sachser N. A Time to Wean? Impact of Weaning Age on Anxiety-Like Behaviour and Stability of Behavioural Traits in Full Adulthood. PloS one. 2016;11(12):e0167652 Epub 2016/12/09. 10.1371/journal.pone.0167652 27930688 PMC5145172

[65] Richter SH, Kästner N, Kriwet M, Kaiser S, Sachser N. Play matters: the surprising relationship between juvenile playfulness and anxiety in later life. Animal Behaviour. 2016;114:261–71. 10.1016/j.anbehav.2016.02.003.

