# Supplementary figures and images for: Post-weaning infant-to-mother bonding in nutritionally independent female mice
Source: PLoS One. 2020 Jan 15;15(1):e0227034. doi: 10.1371/journal.pone.0227034 (PMC6961874; doi:10.1371/journal.pone.0227034)

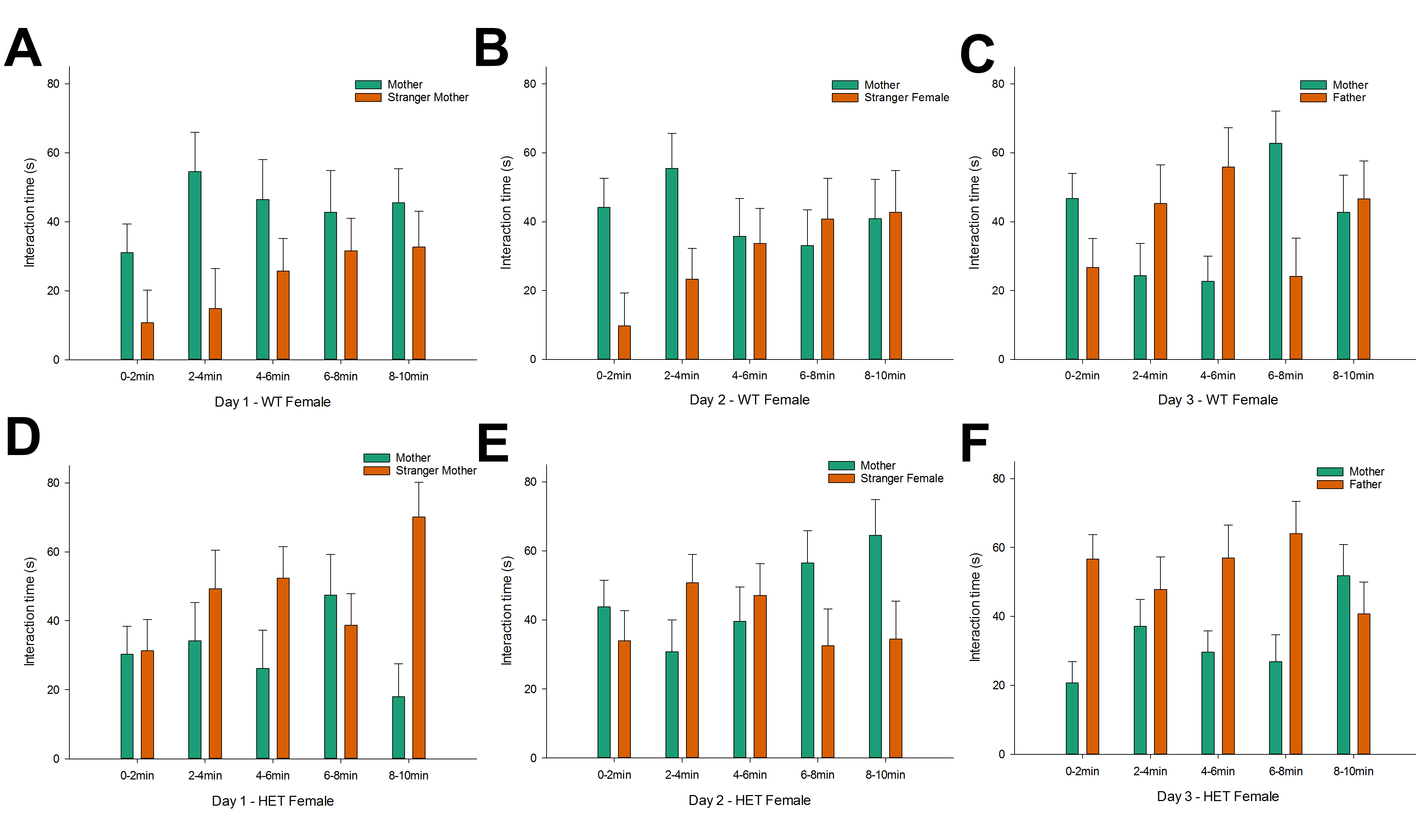

Supplement: S1 Fig — Interaction time per 2 min with the mother (green bars) and other stimulus mice (orange bars) per testing day in female control mice and female Nbea+/- mice. (A-C) Female controls initially prefer contact with their mother in comparison to other stimulus mice. This marked preference declines across testing days. (D-F) Female Nbea+/- mice showed increased interaction with the stranger mother in comparison with their own mother. Reduced interaction with the mother was less pronounced during the second day, but was intensified when the father was presented on the third day of testing. All data are presented as mean + SEM (control females (n = 10) and Nbea+/- females (n = 12)). Abbreviations: WT = wildtype control; HET = heterozygote Nbea+/-. (TIF) [file pone.0227034.s001.TIF]

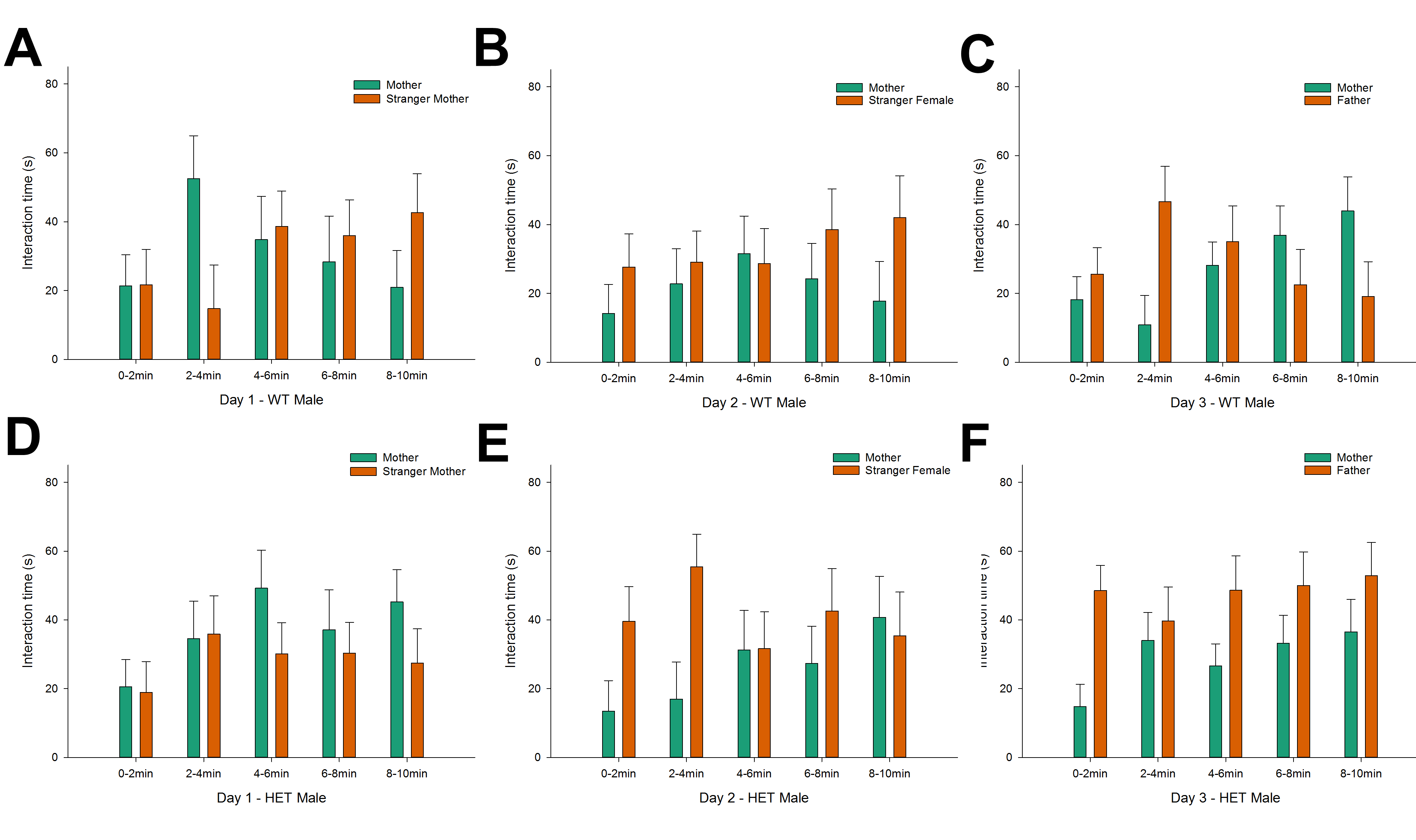

Supplement: S2 Fig — Interaction time per 2 min with the mother (green bars) and other stimulus mice (orange bars) per testing day in male control mice and male Nbea+/- mice. (A-C) Male controls show an initial increase in contact with their mother in comparison to the stranger mother, but maternal preference quickly declines and is not observed again in subsequent days.(D-F) Male Nbea+/- mice do not show maternal preference in comparison with the stranger mother. Increasing maternal avoidance was observed in subsequent comparisons with the other female and the father. All data are presented as mean + SEM (control males (n = 10), Nbea+/- males (n = 11)). Abbreviations: WT = wildtype control; HET = heterozygote Nbea+/-. (TIF) [file pone.0227034.s002.TIF]
